# Reliability and construct validity of the Ottawa valve collapse scale when assessing external nasal valve collapse

**DOI:** 10.1186/s40463-018-0259-x

**Published:** 2018-02-14

**Authors:** Hedyeh Ziai, James P. Bonaparte

**Affiliations:** 1 Faculty of Medicine, University of Ottawa, Ottawa, ON Canada; 2 Department of Otolaryngology–Head and Neck Surgery, University of Ottawa, 1919 Riverside Drive, Suite 309, Ottawa, ON K1H 7W9 Canada

**Keywords:** Nasal obstruction, Cottle maneuver, Reliability, Nasal airway surgery, Septoplasty

## Abstract

**Background:**

Nasal valve collapse is a common cause of nasal obstruction in otolaryngology practice. Common examination methods, such as the Cottle Maneuver and modified Cottle Maneuver are available. However, these methods are dichotomous and do not provide ordinal severity information. The Ottawa Valve Collapse Scale (OVCS) is a grading system for assessing and easily grading external nasal valve collapse in patients with a septal deviation. The primary objective was to assess the test-retest reliability and construct validity of the OVCS grading scale. A secondary objective was to perform the same assessments on the Cottle Maneuver.

**Methods:**

Patients with a septal deviation who were requesting surgical correction were prospectively enrolled in the study. All patients were assessed using both the Cottle Maneuver and the OVCS by one otolaryngologist at two visits separated by one month. The phi coefficient was calculated to assess the test-retest reliability of the instruments. Results of the Nasal Obstruction Symptom Evaluation (NOSE) Score was compared to determine construct validity.

**Results:**

Ninety-two patients met our inclusion criteria. The phi coefficient was 0.62 for the OVCS and 0.32 for the Cottle Maneuver. The scores on the NOSE instrument were positively associated with the OVCS scores (*p* = 0.01) while there was no association with the Cottle Maneuver (*p* = 047).

**Conclusion:**

This current preliminary analysis suggests that the novel Ottawa Valve Collapse Scale has good test-retest reliability and construct validity. This scale may help clinicians grade external nasal valve collapse in patients with a septal deviation. Future studies are required to determine if this scale assists surgeons in determining which patients need formal nasal valve surgery in addition to a standard septoplasty.

## Background

Nasal obstruction is a common presenting complaint in otolaryngology practice. Nasal valve collapse (NVC) is recognized as a significant contributor to nasal airway obstruction [1–3]. Although there is a wide range of symptom severity, nasal obstruction has been associated with impairment in patients’ quality of life in nearly all domains [4]. Successful corrections can result in significant improvements in patient satisfaction [5].

Nasal valve collapse is often underdiagnosed in patients undergoing septoplasty and if unrecognized, can result in high rates of surgical failure [4–9]. Importantly, NVC often coexists with a septal deviation [10, 11] and the accurate preoperative evaluation and diagnosis of valve dysfunction is an essential requisite for optimal surgical planning to ensure best possible results for patients undergoing nasal airway surgery.

The preoperative diagnosis of nasal valve dysfunction is commonly based on physical examination findings [12]. Particularly, the anatomic areas involving the internal and external valves require examination. Common techniques which aid in the diagnosis include the Cottle Maneuver (CM) and the modified Cottle Maneuver [13–15].

The Cottle Maneuver is the most commonly used test to diagnose clinically relevant nasal valve competency requiring surgical correction [2, 12]. Despite its widespread use, to our knowledge it has never been validated using patient centered outcomes, nor has it been confirmed to be a reliable test [16–18]. Additionally, the test only allows for a dichotomous assessment and does not provide information on valve collapse severity nor does it differentiate between external and internal valve collapse. This limitation was overcome by the modified Cottle Maneuver and can be used to differentiate between internal and external nasal valve collapse [15]. This test may predict surgical outcomes in patients undergoing a functional rhinoplasty; however similar to the CM, it provides dichotomous data for each nasal valve and does not provide an assessment of severity. Additionally, it is unknown if this test has adequate construct validity in patients with a co-morbid septal deviation.

Most [13, 14] divided the nasal sidewall into the upper zone (overlying the traditional area defined as the internal nasal valve) and lower zone (overlying the external nasal valve). The authors developed and validated a 4-point severity scale for the upper zone. However, grading was not provided for the location corresponding to the external nasal valve [14]. Recently, Lindsay et al. [19] developed a useful nasal anatomic worksheet to identify areas of concern in patients with nasal airway obstruction. Although this worksheet demonstrated good reliability, there were no details regarding the specifics of grading scores.

To overcome some of the deficiencies of the other methods of external nasal valve assessment, our team has developed a new scale, the Ottawa Valve Collapse Scale (OVCS). The OVCS allows clinicians to grade external nasal valve collapse under normal physiological conditions without altering the patients’ anatomy. The OVCS is an ordinal scale and thus allows for grading of symptom severity from mild to severe. The scale was developed with considerations of the principles of flow dynamics. Specifically, treating the entire nose as a single system and not two isolated systems (right and left nasal cavities). Modeling the nose in this manner may help to differentiate disease severity in patients with a septal deviation and thus potentially guide treatment decisions.

The principal aim of this paper was to assess the test-retest reliability of the Ottawa Valve Collapse Scale and determine its construct validity. As a secondary objective, we will assess the same outcomes for the Cottle Maneuver.

## Methods

### Patients and treatment

This was a prospective case series of patients presenting to an Otolaryngology- Head and Neck Clinic in Ottawa with nasal obstruction between November 1st, 2014 and November 1st, 2016. This study was approved by our institutional ethics review board (20140735-01H) and all patients consented to inclusion.

Patients were included if they had nasal obstruction caused by a septal deviation (with or without evidence of nasal valve collapse). Patients were enrolled consecutively into the prospective protocol from the clinical practice of the supervising author (JB), a practice focusing in General Otolaryngology and Facial Plastic and Reconstructive Surgery. Inclusion criteria for the study were as follows: patients at least 18 years of age, a septal deviation, symptoms lasting at least 12 months and symptoms continuing after a minimum one-month trial of intranasal corticosteroids. Patients were excluded if they were under the age of 18 years, a Nasal Obstruction Symptom Evaluation (NOSE) [1] score of ≤1, if they had a prior septal or nasal surgery, a known traumatic cause of a septal deviation, nasal obstruction due to allergic rhinitis, chronic, or a neoplastic or autoimmune process.

### Sample size calculation

A sample size calculation was performed to determine the number of patients required to compare differences between grades using an Kruskal-Wallis Test. Assuming a 95% power with a significant difference defined as 5%, we determined a difference in the NOSE score of 2 as significant with a standard deviation of 3. A minimum of 24 patients were required per group.

### Protocol

Patients were assessed at two visits separated by a minimum of 1 month using a standard pre-operative clinical evaluation of the nasal airway. This included the administration of the nasal obstruction symptom evaluation (NOSE) questionnaire, flexible endoscopic examination, the Cottle Maneuver and the Ottawa Valve Collapse Scale. All examinations were performed without decongestant applied. The examiner was blinded to the results of the first visit on the subsequent visit.

To conduct the Cottle Maneuver, the clinician placed their left and right thumb on the cooresponding left and right cheek skin near the alar facial groove on the patients face. The clinician then applied a lateral force with each thumb, thereby lateralizing the cheek soft tissue and adjacent nasal wall. The patient was asked to breathe in through his/her nose at maximum effort. A patient was defined as having a positive CM if they reported subjective improvement in breathing compared to breathing without the CM.

To assess for external nasal valve collapse using the OVCS, patients were asked to breathe in through his/her nose at maximum effort. The maneuver was performed by viewing the nose aided by a light source from the basal view. The caudal edge of the lower lateral cartilage was used as the lateral boundary with the septum used as the medial boundary. Collapse was graded by viewing the degree of collapse and resulting alteration in airflow.

The degree of nasal valve collapse was graded according to the following classification: *Grade 0:* No external nasal valve collapse; *Grade I:* Unilateral Partial Collapse (≤99%); *Grade II:* Bilateral Partial collapse (≤99%) or Unilateral Complete Collapse (100%); or *Grade III:* Bilateral Complete Collapse (100%). Partial collapse was defined as active narrowing of the external nasal valve occurring during deep nasal inspiration without complete airflow blockage or contact with the septum. Complete collapse was defined as complete blockage of airflow and nasal side alar or mucosa overlying the lower lateral cartilage contacting the septum medially.

### Statistical analysis

Statistical analysis was carried out using Minitab 17 (Minitab Inc.). The changes in NOSE score were reported as absolute values (raw scores from the NOSE instrument). The phi coefficient was calculated to assess for test-retest reliability of the instruments. To determine the construct validity, a Kruskal-Wallis Test was performed to compare the results of the NOSE questionnaire between severity grades for each physical examination. A value of *p* < 0.05 was considered statistically significant.

## Results

### Patients

Between November 1st, 2014 and November 1st, 2016, 92 patients consened to the study, a sample of convenience. Thirty patients (33%) were female and 62 (67%) were male. The mean (±SD) age was 40.4 (±15.3) years. The mean (±SD) baseline NOSE score was 14.1 (±4.0).

### Cottle maneuver

Sixty-eight (74%) patients had a positive Cottle result on the initial exam, while 62 (67%) patients had a positive Cottle Maneuver on the second exam. Of these, 52 were Cottle positive on both visits, and 14 were Cottle negative on both visits (Table 1). The remaining patients had discordant examinations (Table 1). The phi coefficient for the CM was 0.32, signifying a weak test re-test reliability.

**Table 1:** Cottle Maneuver Test-Retest Results

		Visit 1 Score		
		Positive	Negative	Total
Visit 2 Score	Positive	52	16	68
	Negative	10	14	24
	Total	62	30	92

### Ottawa valve collapse scale

The results of the OVCS during the two visits are included in Table 2. The phi coefficient for the test-retest reliability of the OVCS was 0.62, signifying a moderate strength positive reliability.

**Table 2:** The Ottawa Valve Collapse Scale Test-Retest Results

	Visit 1 Score				
Visit 2 Score		0	1	2	3
	0	28	7	2	0
	1	5	10	5	0
	2	2	6	20	3
	3	0	0	2	2

### Construct validity

There was no difference in NOSE scores between patients who were positive or negative on the CM (*p* = 0.47) (Table 3). There was a significant difference between NOSE scores between OVCS grades, with higher NOSE scores occurring in higher OVCS grades (*p* = 0.012) (Table 4).

**Table 3:** NOSE Score for the Cottle Maneuver

CM	*n*	Mean	SD	Median
Negative	30	13.8	3.4	15
Positive	66	14.2	4.2	14

*p* = 0.47

**Table 4:** NOSE Score for each grade of the OVCS

OVCS Grade	*n*	Mean	SD	Median
0	30	12.1	4.4	12
1	24	14	3	14
2	34	14.6	4.1	14
3	8	18.2	2.1	19

*p* = 0.012

## Discussion

The Ottawa Nasal Valve Scale was developed to assist clinicians in quickly grading nasal valve collapse in patients with a septal deviation. Although patients with a septal deviation may also have evidence of nasal valve collapse, determining which patients require nasal valve surgery, in addition to a septoplasty, can be difficult. The results of this study demonstrate that as the grade of nasal valve collapse increases, the patients’ symptom severity increases suggesting worsening symptoms with worsening degrees of nasal valve collapse.

Although other methods to assess nasal valve collapse exist, they are often limited as they provide dichotomous data and do not allow for grading of symptom severity. Common tests, such as the CM, is patient-reported and examiner dependent and may explain the low test-retest reliability. Factors such patient biases, non-measurable factors (i.e. current status of the nasal cycle) and clinician factors (i.e. performance of the exam, pressure on the skin) may influence scores. Some of these factors may have been relieved in the OVCS, wherein the physician is assessing the valve collapse in a dynamic situation without any physical alteration of the nose.

Previous studies have attempted to grade nasal valve collapse. Most [13] originally described a three-point grading system similar to our method. This was later validated by Tsao et al. [14] Although the authors use this system to grade the lateral wall collapse, it corresponds to what is commonly described as the area overlying the internal nasal valve and not the external nasal valve. Similar to our method, the authors focused on the dynamic collapse of the nasal sidewall relative to its resting state. The authors noted good inter-rater and intra-rater reliability.

Similarly, Poirrier et al. [20] used a 4-point grading system that was applied to both the left and right side of the nose. Similar to the OVCS, it measured collapse under dynamic situations, but also considered static narrowing. The results of the study were similar to ours; however, the OVCS attempts to treat the nasal cavity as a complete system with one grade for overall breathing as opposed to bilateral scores. Our team believes that it is important to treat the nose as a complete system, particularly when deciding which treatments or combination of treatments are required.

The correct preoperative evaluation of valvular dysfunction is an essential requisite for optimal surgical planning to ensure best possible results for patients undergoing nasal airway surgery. In a retrospective series of patients who presented with failed septoplasty, 51% had significant NVC [9]. Missed preoperative dynamic nasal valve collapse has been suggested as the most common cause of septoplasty failure [1, 21–23]. A recent study suggested that nasal valve dysfunction should be considered in all patients with a septal deviation undergoing septoplasty [5]. However, as noted by Schalek and Hahn [24], unilateral nasal valve collapse without bilateral collapse is adequately corrected by a septoplasty alone if a septal deviation is present, while patients with evidence of bilateral collapse are likely more resistant to a single treatment. The OVCS aims to differentiate these patients and assist with determining which patients require nasal valve repair at the time of a septoplasty.

The NOSE questionnaire was selected as it is the most widely used validated questionnaire; [12] it is a responsive and disease-specific assessment tool that can quantitatively assess nasal obstructive symptoms in our study cohort [4, 5, 25–27]. The NOSE instrument been recently used in patients undergoing nasal valve correction surgery in evaluating for subjective improvements in nasal obstructive symptoms [7, 8, 28–31].

Ultimately, our aim was to develop a scale that may be used to grade external nasal valve dysfunction with the future goal of allowing clinicians to predict which patients may benefit from a more extensive nasal airway surgery including nasal valve correction. A simple tool may be the most practical in the clinical setting; however, there is no study that demonstrates which outcome measure (or combination of outcome measures) may accurately and precisely identify those patients who may benefit from nasal valve surgery, particularly when other comorbid diagnosis (septal deviation) are present. Those with more severe scores may benefit from more extensive nasal airway surgery, including nasal valve correction surgery, however future studies are required to assess this.

Although this study achieved both of its objectives, there were some limitations and important points to consider. The goal of this paper was to assess the validity and reliability of the OVCS in terms of patient symptom severity and not its ability to predict which patients require specific surgical procedures. However, this current study should serve as a basis for future research that may use the OVCS to diagnose and grade nasal valve collapse. In turn, this research should evaluate if the OVCS assists in determining which patients may require nasal valve correction surgery in addition to a septoplasty. This is important as it remains unclear if the use of the OVCS may help direct surgical planning and lead to improved patient outcomes. In addition to this, future research should assess whether combination of physical examination (ie OVCS, Cottle Maneuver, Modified Cottle) will result in the ability to predict what surgical procedure or combination of procedures are required. Future efforts may also focus on assessing criterion validity of the scale with evaluations of nasal valve collapse including objective measurement of objective nasal airflow.

The median follow-up time between the two visits was 2 months. There may have changes in patients’ symptoms that may have underestimated the reliability of the instruments. That being said, the range of follow-up was random and thus we believe the error associated with this would also be random and not bias our findings. This study’s findings are based on a single surgeon’s assessments who is also involved in the development of the Ottawa Valve Collapse Scale. Although the surgeon was blinded to the results of the first visit, there may have been some element of recall bias and/or situational bias. Furthermore, there is no gold standard for determining NVC symptoms, and thus we used the nasal obstruction score as determined by the NOSE [32] instrument to determine the construct validity. In addition, this present study was performed on English-speaking Canadians in a single tertiary care center in Ottawa, and may limit its application to other populations. Finally, although the scale was based on application of flow dynamic principles, there was no confirmation using objective measures.

Perhaps most importantly, at this point only one physician has performed an assessment of test-retest and construct validity. Although the study met our stated objectives, we are currently in the process of completing a study assessing the inter-rater reliability as well as a multi-rated assessment of intra-rater reliability of the scale.

## Conclusion

To the best of our knowledge, the present study is the first attempt to ascertain the test-retest reliability of the Cottle Maneuver and the OVCS, and the construct validity of the maneuver in assessing nasal obstruction symptoms. This current preliminary analysis suggests that the novel Ottawa Valve Collapse Scale may have improved test-retest reliability when compared to the Cottle Maneuver for diagnosing nasal valve collapse, and improved validity for patient-reported nasal obstruction. The OVCS may serve as a means in the future in diagnosing nasal valve collapse in patients undergoing nasal airway surgery.

## Abbreviations

CM: Cottle Maneuver
NOSE: Nasal Obstruction Severity Evaluation
NVC: Nasal Valve Collapse
OVCS: Ottawa Valve Collapse Scale
